# Supplementary material for: Hyaluronic Acid in Synovial Fluid Prevents Neutrophil Activation in Spondyloarthritis
Source: Int J Mol Sci. 2023 Feb 4;24(4):3066. doi: 10.3390/ijms24043066 (PMC9964069; doi:10.3390/ijms24043066)
Supplement: Supplementary file 1 [file ijms-24-03066-s001.zip › ijms-2130667-supplementary.pdf]

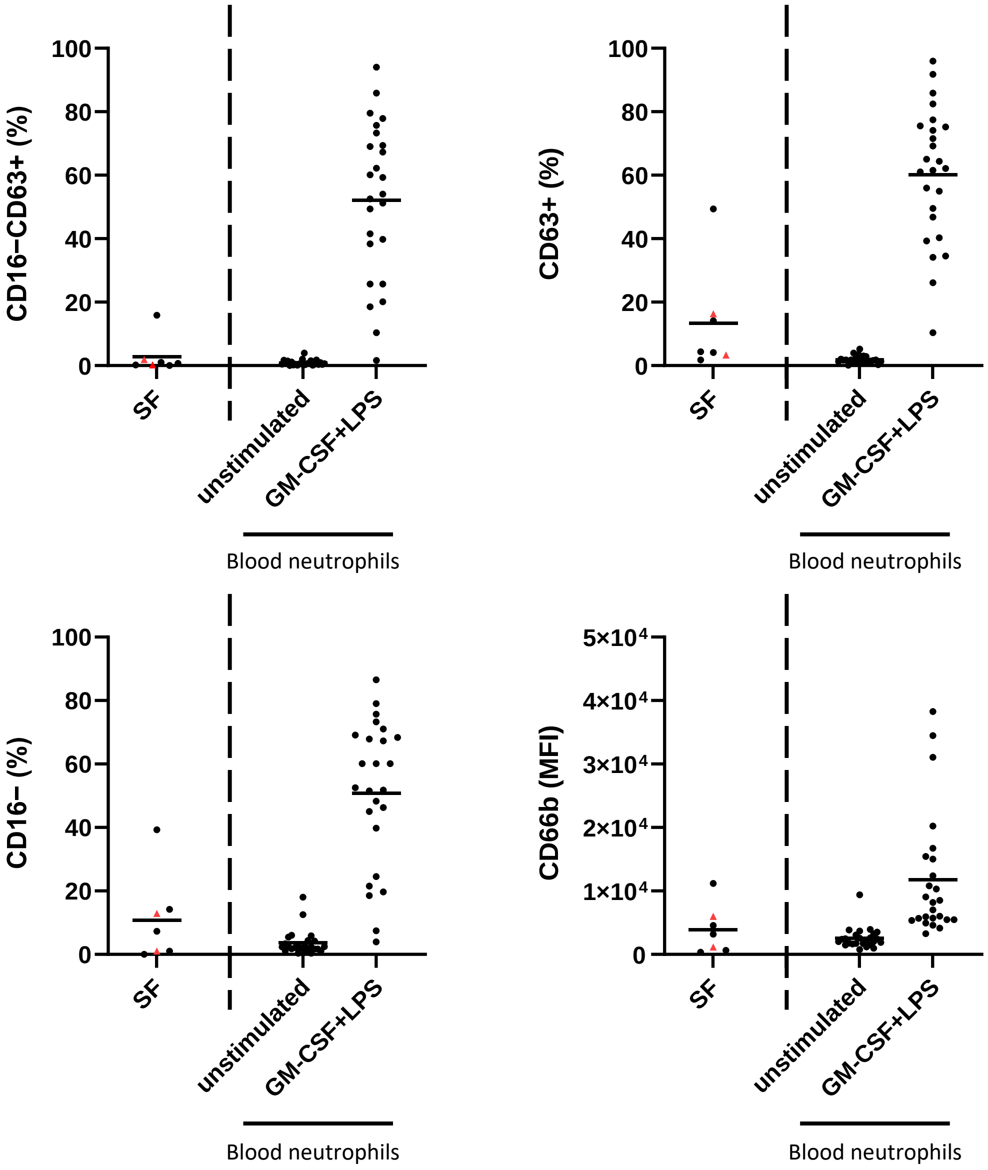

Figure S1. Neutrophils derived from SF of RA and OA patients do not degranulate. Neutrophils from SF from RA patients (black circles (n = 5)) and OA patients (red triangles (n = 2)) were analyzed by flow cytometry and compared to neutrophils derived from blood. Full neutrophil degranulation as measured by percentage of CD16−CD63+ neutrophils, secretory vesicle degranulation as measured by percentage of CD16- neutrophils, azurophillic degranulation as measured by percentage of CD63+ neutrophils, and specific and gelatinase degranulation as measured as mean fluorescent intensity (MFI) of CD66b. Data are presented as mean and individual points.

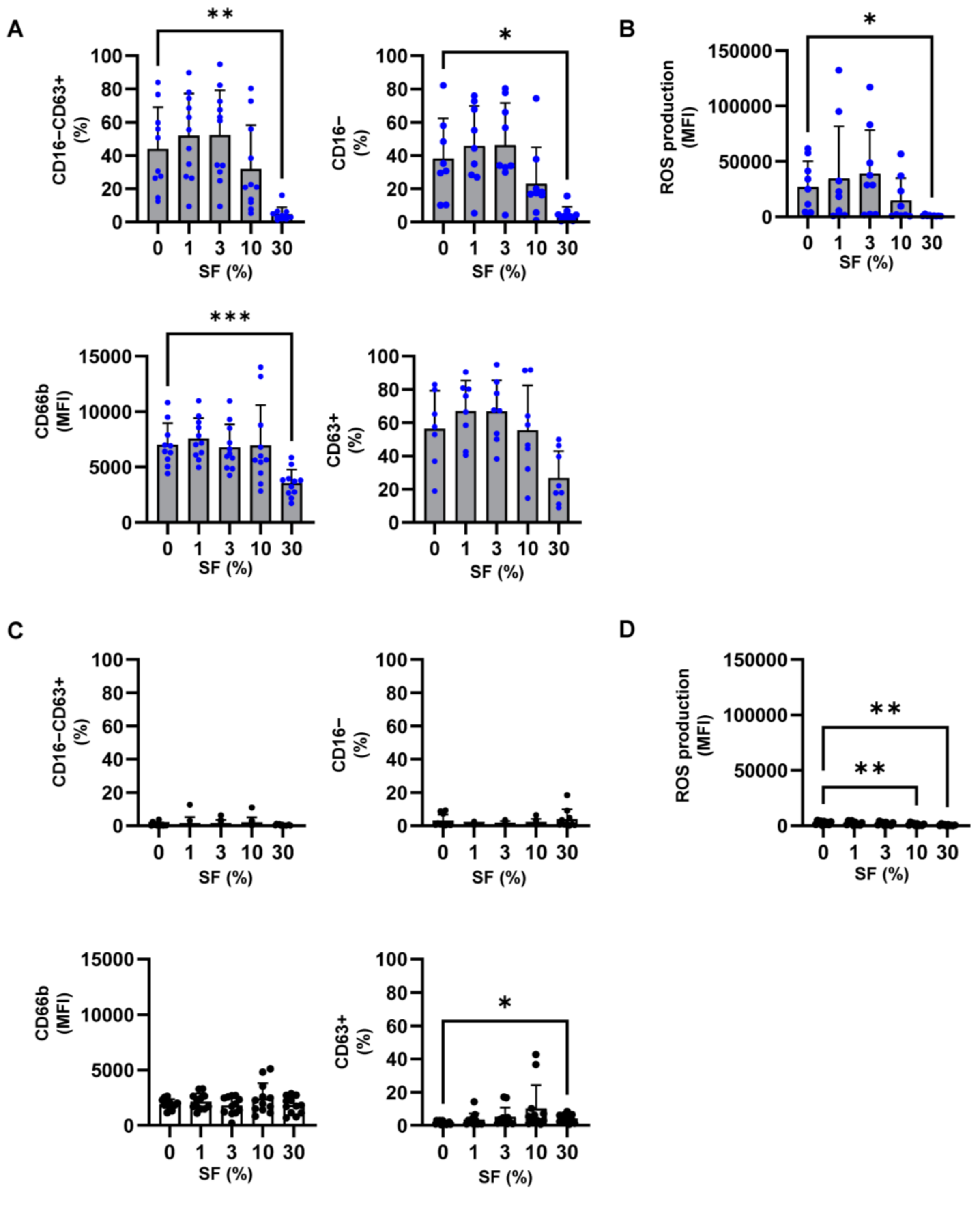

Figure S2. Titrations of SF on stimulated and unstimulated neutrophils. (**A**) Blood-derived neutrophils from healthy donors (n = 11–14) were cultured for 2 hours in the presence of GM-CSF (50 U/mL) and LPS (10 ng/mL) and with various concentrations of SF of SpA patients (n = 9–12, pat# 7, 8, 10, 12–20). Percentage of CD16−CD63+, CD16−, CD63+, and MFI of CD66b membrane expression as a measure for degranulating neutrophils. (B) Blood-derived neutrophils from healthy donors (n = 9) were cultured for 1 hour in the presence of 123-DHR and GM- CSF (50 U/mL) and LPS (10 ng/mL) and with various concentrations of SF of SpA patients (n = 6, pat# 13, 14, 16–19). Percentage of ROS production. (**C**) Blood-derived neutrophils from healthy donors (n = 11) were cultured for 2 hours with various concentrations of SF from SpA patients (n = 9, pat# 12–20). Percentage of CD16−CD63+, CD16−, CD63+, and MFI of CD66b membrane expression as a measure for degranulating neutrophils. n = 11. (D) Blood-derived neutrophils from healthy donors (n = 10) were cultured for 1 hour in the presence of 123-DHR and with various concentrations of SF of SpA patients (n = 6, pat# 13, 14, 16–19). Percentage of ROS production. Data are presented as mean ± SD. * $p < 0.05$, ** $p < 0.01$, and *** $p < 0.001$. The p-values were calculated using a one-way ANOVA.

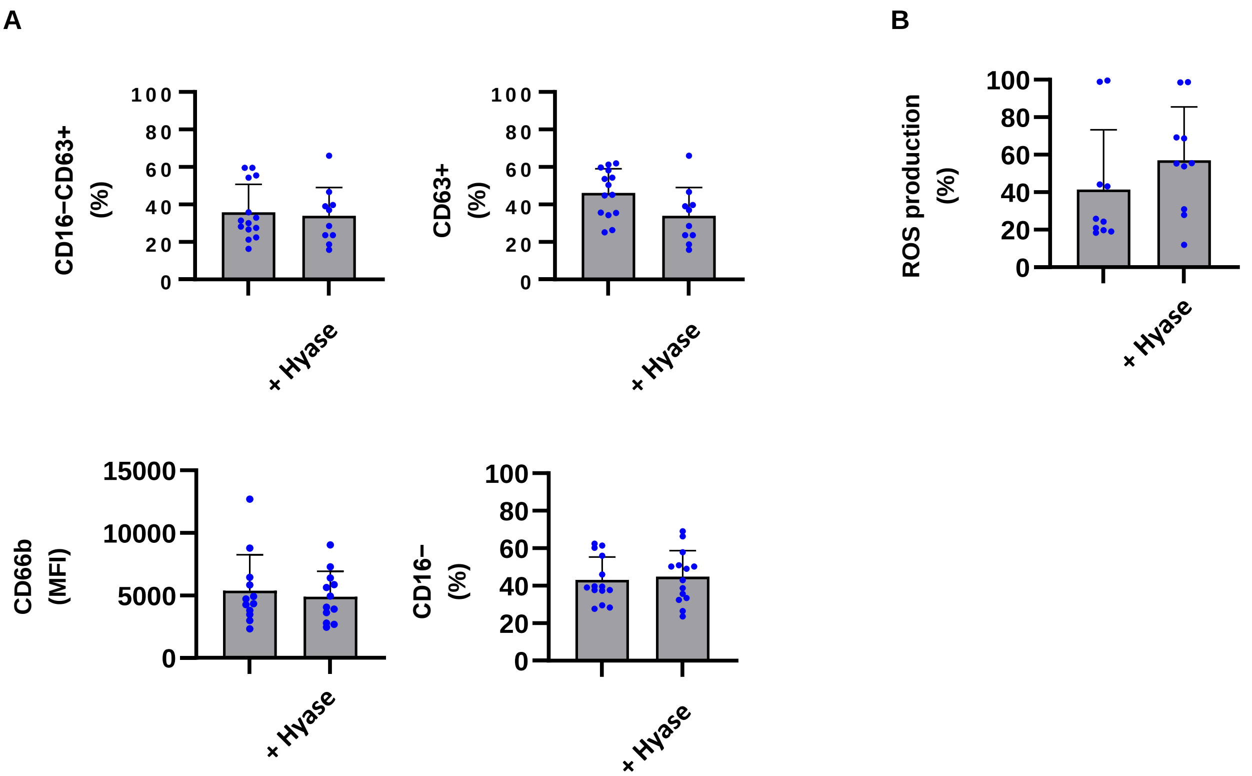

Figure S3. Hyase has no effects on neutrophil activation. (**A**) Blood-derived neutrophils were cultured for 2 hours in the presence of GM-CSF (50 U/mL) and LPS (10 ng/mL), with or without hyase. Percentage of CD16-CD63+, CD16-, CD63+, and MFI of CD66b membrane expression as a measure for degranulating neutrophils. n = 11–19 (**B**) Blood-derived neutrophils were cultured for 1 hour in the presence of 123-DHR and GM-CSF (50 U/mL) and LPS (10 ng/mL), with or without hyase. Percentage of ROS production. n = 10. Data are presented as mean ± SD.

Table S1. Description of the patient cohort and description of SF data from OA and RA patients. ND = not determined.

Patient #	Diagnosis	Gender M/F	Age Years	Treatment	Type of bDMARd (ia)	Total Cells in SF (cells/mL)	Amount of Neutrophils in SF (%)
21	RA	F	85	NSAID		0.3×10^6	25.0
22	RA	M	67	None		ND	ND
23	RA	M	36	None		10.7×10^6	58.8
24	OA	M	59	NSAID, csDMARD, bDMARD	anti-TNF	3.2×10^6	21.0
25	OA	M	60	csDMARD, bDMARD	anti-TNF	ND	ND
26	RA	F	50	Steroid		9×10^6	21.6
27	RA	M	37	None		ND	ND
